# Supplementary material for: Primary Founder Mutations in the PRKDC Gene Increase Tumor Mutation Load in Colorectal Cancer
Source: Int J Mol Sci. 2022 Jan 6;23(2):633. doi: 10.3390/ijms23020633 (PMC8775830; doi:10.3390/ijms23020633)
Supplement: Supplementary file 1 [file ijms-23-00633-s001.zip › ijms-1448683-supplementary.pdf]

Supplementary Materials

Primary Founder Mutations in the PRKDC Gene Increase Tumor Mutation Load in Colorectal Cancer

Hajnalka Laura Pálinkás [1,2], Lőrinc Pongor[3,4], Máté Balajti [3], Ádám Nagy [3,4], Kinga Nagy [1,2], Angéla Békési [1,2], Giampaolo Bianchini [5], Beáta G. Vértessy [1,2,*] and Balázs Győrffy [3,4,*]

[1] Genome Metabolism Research Group, Institute of Enzymology, Research Centre for Natural Sciences, Magyar tudósok körútja 2., H-1117 Budapest, Hungary; palinkas.hajnalka@ttk.hu (H.L.P.); nagy.kinga@ttk.hu (K.N.); bekesi.angela@ttk.hu (A.B.); vertessy.beata@ttk.hu (B.G.V.)

[2] Department of Applied Biotechnology and Food Sciences, BME Budapest University of Technology and Economics, Szt Gellért tér 4, H-1111 Budapest, Hungary

[3] TTK Lendület Cancer Biomarker Research Group, Institute of Enzymology, Research Centre for Natural Sciences, Magyar tudósok körútja 2., H-1117 Budapest, Hungary; pongorlorinc@gmail.com (L.P.); balajtimate@gmail.com (M.B.); nagy.adam@ttk.hu (Á.N.)

[4] Semmelweis University, Department of Bioinformatics and 2nd Department of Pediatrics, H-1094, Tuzolto u. 7-9, Budapest, Hungary

[5] Department of Medical Oncology, San Raffaele Scientific Institute, via Olgettina 60, 20132 Milan, Italy

* Correspondence: vertessy.beata@ttk.hu (B.G.V.); gyorffy.balazs@ttk.hu (B.G.);

Supplemental Figure S1. Survival analysis in colorectal cancer patients. Forest plot showing survival in patients stratified by the expression levels for the DNA repair pathway signatures **(A)**, Kaplan-Meier survival plot for PRKDC **(B),** and BRCA2 **(C),** and using stratification via the mean expression of all DNA-repair genes **(D)**.

(A)

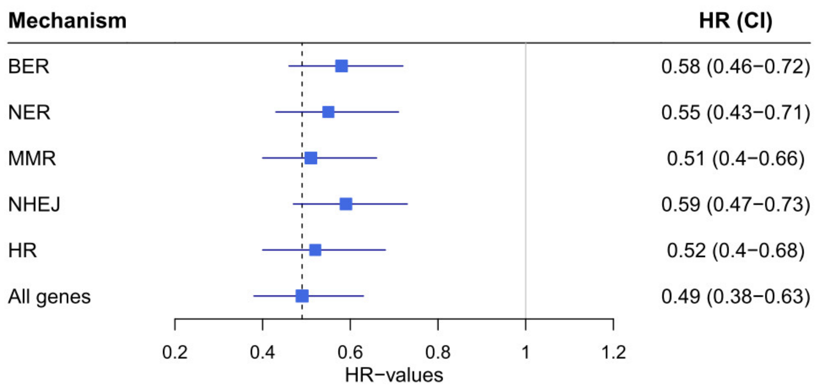

(B) PRKDC

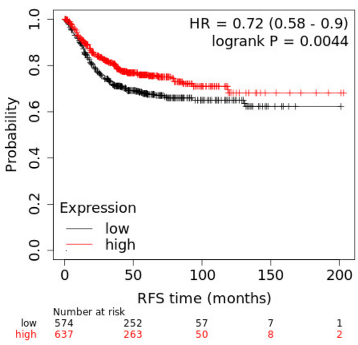

(C) BRCA2

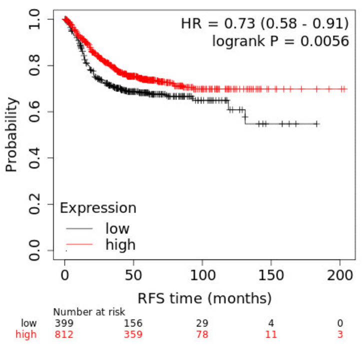

(D)

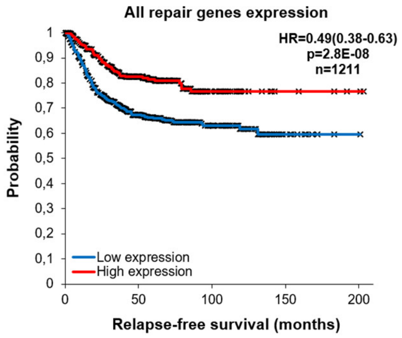

Supplemental Table S1. Genes participating in eukaryotic DNA repair including base-excision repair (BER), mismatch repair (MMR), nucleotide-excision repair (NER), homologous recombination (HR), and non-homologous end joining (NHEJ).

BER		
gene	*also known as*	*function*
UNG	UNG1; UNG2; UDG; DGU; HIGM4; HIGM5; UNG15	Monofunctional glycosylases
MUTYH	MYH	
MBD4	MED1	
TDG	hTDG	
MPG	AAG; MDG; ADPG; APNG; Mid1; anpg; PIG11; PIG16; CRA36.1	
SMUG1	FDG, HMUDG, UNG3	
OGG1	HMMH; MUTM; OGH1; HOGG1	Bifunctional glycosylases
NTHL1	FAP3; NTH1; OCTS3; hNTH1	
NEIL1	FPG1, NEI1, hFPG1	
NEIL2	NEH2, NEI2	
NEIL3	FGP2, FPG2, NEI3, ZGRF3, hFPG2, hNEI3	
APE1	APEX1, APE, APEN, APEX, APX, HAP1, REF1	AP-endonuclease
APE2	APEX2, APEXL2, XTH2, ZGRF2	
XRCC1	RCC	DNA synthesis and ligation
Pol β		
FEN1	MF1, RAD2	
HMGB1	HMG-1, HMG1, HMG3, SBP-1	
PNKP	Q96T60, PNK	
PCNA	ATLD2	
RPA4	HSU24186	
RPA1	P27694	
RPA2	P15927	
RPA3	P35244	
Pol δ	polD	
polD2	P49005	
polD3	Q15054	
polD4	Q9HCU8	
RFC1	A1; RFC; PO-GA; RECC1; MHCBFB; RFC140	
RFC2	P35250	

RFC3	P40938	
RFC4	P35249	
RFC5	P40937	
Polε	polE	
polE2	P56282	
polE3	Q9NRF9	
polE4	Q9NR33	
Pol λ		error prone polymerases for translesion synthesis
polI	Q9UNA4	
polH	Q9Y253	
LIG3	P49916, lig2	ligation
LIG1		
SSBP1	SSBP; mtSSB; Mt-SSB; SOSS-B1	mitochondrial BER
polG1	P54098	
polG2	Q9UHN1	
**NER**		
gene	*also known as*	*function*
RBX1	ROC1; RNF75; BA554C12.1	Damage recognition
CUL4B	MRXHF2, MRXS15, MRXSC, SFM2	
CUL4A	Q13619	
DDB1	XPE; DDBA; XAP1; XPCE; XPE-BF; UV-DDB1	
DDB2	XPE; DDBB; UV-DDB2	
XPC	RAD4, XP3, XPCC, p125	
RAD23A	HR23A, HHR23A	
RAD23B	p58; HR23B; HHR23B	
CETN2	CALT; CEN2	
ERCC8	CKN1, CSA, UVSS2	
ERCC6	CKN2, CSB, UVSS1, COFS, COFS1, POF11, RAD26, ARMD5	
Cdk7	CAK, CAK1, CDKN7, HCAK, MO15, STK1, p39MO15	DNA unwinding
MNAT-1	CAP35, MAT1, RNF66, TFB3	
CCNH	CAK, CycH, p34, p37	
ERCC3	XPB, BTF2, GTF2H, RAD25, TFIIH, TTD2	
ERCC2	XPD; XPDC, COFS2, EM9, TFIIH, TTD, TTD1	

GTF2H5	TTD; TFB5; TTD3; TTDA; TFIIH; TTD-A; TGF2H5; C6orf175; bA120J8.2	
GTF2H1	P62; BTF2; TFB1; TFIIH	
GTF2H2	p44; BTF2; TFIIH; BTF2P44; T-BTF2P44	
GTF2H3	P34; BTF2; TFB4; TFIIH	
GTF2H4	p52; TFB2; TFIIH	
XPA	XP1; XPAC	
RPA4	HSU24186	ssDNA stabilization
RPA1	P27694	
RPA2	P15927	
RPA3	P35244	
ERCC5	XPG; XPGC; UVDR; COFS3; ERCM2; ERCC5-201	Incision
ERCC4	XPF; RAD1; FANCQ; XFEPS; ERCC11	
ERCC1	COFS4, RAD10, UV20	
SLX4	Q8IY92	
PCNA	ATLD2	DNA synthesis and ligation
RFC1	A1; RFC; PO-GA; RECC1; MHCBFB; RFC140	
RFC2	P35250	
RFC3	P40938	
RFC4	P35249	
RFC5	P40937	
Pol δ		
polD2	P49005	
polD3	Q15054	
polD4	Q9HCU8	
polK	Q9UBT6	
Polε		
polE2	P56282	
polE3	Q9NRF9	
polE4	Q9NR33	
LIG3	P49916, lig2	
Lig1		
**MMR**		
gene	*also known as*	*function*

MSH2	FCC1; COCA1; HNPCC; LCFS2; HNPCC1	Mismatch recognition
MSH6	GTBP; HSAP; p160; GTMBP; HNPCC5	
MSH3	DUP; FAP4; MRP1	
MLH3	HNPCC7	recognition, processing, excision
PMS2	MLH4; PMSL2; HNPCC4; PMS2CL	
MLH1	FCC2; COCA2; HNPCC; hMLH1; HNPCC2	
ExoI	HEX1; hExoI	Excision
SLX4	Q8IY92	
RPA4	HSU24186	DNA synthesis and ligation
RPA1	P27694	
RPA2	P15927	
RPA3	P35244	
Pol δ		
polD2	P49005	
polD3	Q15054	
polD4	Q9HCU8	
RCF1	A1; RFC; PO-GA; RECC1; MHCBFB; RFC140	
PCNA	ATLD2	
RFC2	P35250	
RFC3	P40938	
RFC4	P35249	
RFC5	P40937	
Lig1		
polH	Q9Y253	error prone synthesis
polG1	P54098	mitochondrial DNA repair synthesis
polG2	Q9UHN1	
**NHEJ**		
gene	*also known as*	*function*
XRCC6	ML8; KU70; TLAA; CTC75; CTCBF; G22P1	End binding
XRCC5	KU80; KUB2; Ku86; NFIV; KARP1; KARP-1	
PRKDC	HYRC; p350; DNAPK; DNPK1; HYRC1; IMD26; XRCC7; DNA-PKcs	
Rad50	NBSLD; RAD502; hRad50	End processing
NBN	ATV; NBS; P95; NBS1; AT-V1; AT-V2	

Mre11	ATLD; HNGS1; MRE11; MRE11A; MRE11B	
DCLRE1C	SCIDA; SNM1C; A-SCID; RS-SCID; DCLREC1C	
PNKP	Q96T60, PNK	
FEN1	MF1; RAD2; FEN-1	Gap filling
Pol λ		
Pol μ		
DNTT	TDT	
polQ	O75417	
Lig4		Ligation
XRCC4	SSMED	Ligase accessory factor
NHEJ1	XLF	End-joining factor
**HR**		
gene	*also known as*	*function*
SSBP1	SSBP; mtSSB; Mt-SSB; SOSS-B1	recognition, resection and single stranded DNA binding
ATM	AT1, ATA, ATC, ATD, ATDC, ATE, TEL1, TELO1	
Rad50	NBSLD; RAD502; hRad50	
Mre11	ATLD; HNGS1; MRE11; MRE11A; MRE11B	
NBN	ATV; NBS; P95; NBS1; AT-V1; AT-V2	
RBBP8	COM1, CTIP, JWDS, RIM, SAE2, SCKL2	
ATR	Q13535	
TOPBP1	TOP2BP1	
BARD1		
BRCA1	BRCAI, BRCC1, BROVCA1, FANCS, IRIS, PNCA4, PPP1R53, PSCP, RNF53	
BRIP1	BACH1, FANCJ, OF	
FAM175A	ABRA1, CCDC98	
UIMC1	RAP80, X2HRIP110	
BABAM1	C19orf62, HSPC142, MERIT40, NBA1	
BRE	BRCC4, BRCC45	
BRCC3	BRCC36, C6.1A, CXorf53	
PALB2	FANCN, PNCA3	
ExoI	HEX1; hExoI	extension of the resection
BLM	BS; RECQ2; RECQL2; RECQL3	
DNA2	P51530	

HELB	Q8NG08	negative regulator of extended resection
RPA1	P27694	single strand DNA binding
RPA2	P15927	
RPA3	P35244	
RPA4	HSU24186	
Rad51	RECA; BRCC5; FANCR; MRMV2; HRAD51; RAD51A; HsRad51; HsT16930	Filament formation
Rad52		
BRCA2	FAD; FACD; FAD1; GLM3; BRCC2; FANCD; PNCA2; FANCD1; XRCC11; BROVCA2	
SHFM1	ECD; DSS1; SEM1; SHFD1; SHSF1; Shfdg1	
SYCP3	COR1; SCP3; SPGF4; RPRGL4	
XRCC3	O43542	
RAD51C	O43502	
XRCC2	O43543	
RAD51B	O15315	
RAD51D	O75771	
Rad54B	RDH54	Strand invasion
Pol δ		DNA synthesis
polD2	P49005	
polD3	Q15054	
polD4	Q9HCU8	
RCF1	A1; RFC; PO-GA; RECC1; MHCBFB; RFC140	
PCNA	ATLD2	
RFC2	P35250	
RFC3	P40938	
RFC4	P35249	
RFC5	P40937	
polZ	O60673	translesion synthesis, error-prone
Rev7	Q9UI95	
REV1	Q9UBZ9	
polH	Q9Y253	
SLX4	Q8IY92	resolution of Holliday junction

Eme1	MMS4L; SLX2A	
Mus81	SLX3	
TOP3A	TOP3; ZGRF7	
RMI1	Q9H9A7	
RMI2	Q96E14	
MLH3	HNPCC7	
MLH1	FCC2; COCA2; HNPCC; hMLH1; HNPCC2	
FAN1	Q9Y2M0	
GEN1	Q17RS7	
LIG1		ligation
